# Iron Oxide Incorporated Conjugated Polymer Nanoparticles for Simultaneous Use in Magnetic Resonance and Fluorescent Imaging of Brain Tumors

**DOI:** 10.3390/pharmaceutics13081258

**Published:** 2021-08-14

**Authors:** Nuria Arias-Ramos, Luis Exequiel Ibarra, María Serrano-Torres, Balbino Yagüe, Matías Daniel Caverzán, Carlos Alberto Chesta, Rodrigo Emiliano Palacios, Pilar López-Larrubia

**Affiliations:** 1 Instituto de Investigaciones Biomédicas “Alberto Sols”, Consejo Superior de Investigaciones Científicas-Universidad Autónoma de Madrid (CSIC-UAM), 28029 Madrid, Spain; narias@iib.uam.es (N.A.-R.); maria.serranotorres@estudiante.uam.es (M.S.-T.); byague@iib.uam.es (B.Y.); 2 Instituto de Biotecnología Ambiental y Salud (INBIAS), Universidad Nacional de Río Cuarto (UNRC) y CONICET, Córdoba X5800BIA, Argentina; 3 Departamento de Biología Molecular, Facultad de Ciencias Exactas, Fisicoquímicas y Naturales, Universidad Nacional de Río Cuarto (UNRC), Córdoba X5800BIA, Argentina; dcaverzan@ayv.unrc.edu.ar (M.D.C.); 4 Instituto de Investigaciones en Tecnologías Energéticas y Materiales Avanzados (IITEMA), Universidad Nacional de Río Cuarto y CONICET, Córdoba X5800BIA, Argentina; cchesta@exa.unrc.edu.ar (C.A.C.); rpalacios@exa.unrc.edu.ar (R.E.P.); 5 Departamento de Química, Facultad de Ciencias Exactas, Fisicoquímicas y Naturales, UNRC, Córdoba X5800BIA, Argentina

**Keywords:** theranostic, conjugated polymer, iron oxide nanoparticles, glioblastoma, preclinical trials, nanoparticles, MRI

## Abstract

Conjugated polymer nanoparticles (CPNs) have emerged as advanced polymeric nanoplatforms in biomedical applications by virtue of extraordinary properties including high fluorescence brightness, large absorption coefficients of one and two-photons, and excellent photostability and colloidal stability in water and physiological medium. In addition, low cytotoxicity, easy functionalization, and the ability to modify CPN photochemical properties by the incorporation of dopants, convert them into excellent theranostic agents with multifunctionality for imaging and treatment. In this work, CPNs were designed and synthesized by incorporating a metal oxide magnetic core (Fe_3_O_4_ and NiFe_2_O_4_ nanoparticles, 5 nm) into their matrix during the nanoprecipitation method. This modification allowed the in vivo monitoring of nanoparticles in animal models using magnetic resonance imaging (MRI) and intravital fluorescence, techniques widely used for intracranial tumors evaluation. The modified CPNs were assessed in vivo in glioblastoma (GBM) bearing mice, both heterotopic and orthotopic developed models. Biodistribution studies were performed with MRI acquisitions and fluorescence images up to 24 h after the i.v. nanoparticles administration. The resulting IONP-doped CPNs were biocompatible in GBM tumor cells in vitro with an excellent cell incorporation depending on nanoparticle concentration exposure. IONP-doped CPNs were detected in tumor and excretory organs of the heterotopic GBM model after i.v. and i.t. injection. However, in the orthotopic GBM model, the size of the nanoparticles is probably hindering a higher effect on intratumorally T2-weighted images (T_2_WI) signals and T_2_ values. The photodynamic therapy (PDT)—cytotoxicity of CPNs was not either affected by the IONPs incorporation into the nanoparticles.

## 1. Introduction

Malignant gliomas and more specifically glioblastomas (GBM), the most common primary tumor of the central nervous system in adults, are considered as one of the most highly destructive brain diseases and the deadliest of human cancers [1]. These types of tumors seem to be more frequent, not only because of a true rise in their incidence, but also due to the increase in life expectancy of people and scientific and technological advances that allow their early diagnosis [2,3]. Despite the great advance in the understanding of the disease over the last decades, the current medical management of GBM is still based on traditional approaches such as the maximum safe resection followed by radiotherapy and concomitant and adjuvant chemotherapy with temozolomide. Additionally, disease relapse occurs inevitably nearly two years after these treatments and is a major concern [4,5], therefore, it is necessary to improve treatment and early validation of therapies. Nanotechnology could be the source of promising therapeutic nanoparticles (NPs) for effective mitigation or complete cure of the disease. In fact, over the past two decades, considerable efforts have been devoted to that and many research groups have reported the development of different types of theranostic nanoparticles for GBM treatment [6]. In this sense, hybrid nanoparticles which incorporate multiple functionalities represent one of the most promising option mainly due to their combined ability to label and kill tumor cells [4,6]. Aimed to this, our group has recently developed metallated porphyrin-doped conjugated polymer nanoparticles (CPNs) for highly efficient photodynamic therapy (PDT), a therapeutic approach of growing interest in the treatment of GBM and the prevention of local tumor recurrence [7]. These CPNs have been shown to be effective at eliminating glioma tumor cells through ROS-induced apoptotic damage thus highlighting their potential use in photo-assisted treatment of GBM [8,9,10]. Owing to their excellent light-harvesting and photoemission properties combined with their amenable surface-functionalization to target specific cells, CPNs have been successfully applied as target-specific labels for both in vitro and in vivo fluorescence imaging and identification of cancer cells. Additionally, by incorporating carefully chosen molecular dopants into CPNs their capacity as photosensitizers for singlet oxygen (^1^O_2_) production can be greatly increased and applied for tumor reduction/elimination [8,11]. 

Despite the potentiality of these nanoparticles in cancer treatment, preclinical information regarding CPN pharmacokinetic, biodistribution, overall safety upon systemic application and particularly using GBM models in animals, is scarce [9,12,13]. To evaluate some of these parameters, in vivo imaging techniques, like fluorescence imaging and magnetic resonance imaging (MRI), can be highly informative. MRI is a well-known technology used for the evaluation of intracranial lesions, including gliomas, in clinical and pre-clinical set-ups [14,15]. MRI signal intensity in vivo is related, among other parameters, to the relaxation times T_1_ (spin-lattice relaxation) and T_2_ (spin-spin relaxation) of water protons, and incorporation of metallic centers, such as iron oxide nanoparticles (IONPs) into NPs, results in hybrid nanoconstructs with excellent T_2_ contrast enhancement [16,17,18]. Recently, fluorescence-guided surgery, with small molecule-based fluorophores and some others based on nanoparticle systems, has emerged as a rapid and cost-effective alternative to accurately visualize neoplastic areas in real-time to guide resection of GBM [19,20,21]. For that reason, fluorescent-metallic hybrid nanoparticles have been of particular interest in the development of diagnostic and therapeutic materials [22,23,24,25]. Also, a few efforts have been made to develop CPNs incorporating IONPs as potential bimodal imaging agents [23,26], however, little is known about in vivo imaging performance in particular in GBM models. Preclinical GBM models depending on where they are generated (orthotopic vs. heterotopic), they can involve the blood-brain barrier (BBB) as one of the variables to determine the success in penetration and intratumoral accumulation of the vast majority of nanoparticles currently under investigation. In addition, the evaluation of biodistribution and tumor accumulation for CPNs based on poly(9,9-dioctylfluorene-alt-benzothiadiazole (F8BT) could be followed using fluorescence imaging, however the fluorescence signal can be affected by the biological environment due to other natural fluorochromes that emit at shorter wavelength giving misinterpreted results [27]. Contemplating this, the validation of fluorescence data, especially in short-wavelength with other imaging technology is of particular interest for this type of tumor [28]. With this aim, in this study, we report the synthesis and evaluation of CPN incorporating variable amounts of IONPs (CPN-IONPs). The resulting NPs size, morphology, photophysics and MRI contrast performance were characterized. Biocompatibility in GBM tumor cell cultures, and biodistribution and uptake in GBM tumor bearing mice was also evaluated to determinate the intratumoral accumulation of IONP-doped CPNs.

## 2. Materials and Methods

### 2.1. Materials

The fluorescent conjugated polymer (CP) poly(9,9-dioctylfluorene-alt-benzothiadiazole) (F8BT, Mn = 70,000 g/mol, PDI = 2.4, ADS Inc., Saint-Elzéar, QC, Canada), the comb-like polymer, polystyrene grafted with ethylene oxide functionalized with carboxyl groups (PS-EG-COOH, backbone Mn = 6500 g/mol, branches of Mn = 4600 g/mol, Polymer Source Inc., Dorval, QC, Canada), the IONPs based on Fe_3_O_4_ (Mn = 231.54 g/mol) and NiFe_2_O_4_ (Mn = 234.38 g/mol) (5 nm diameter) capped with oleic acid (~2 nm thickness) (LiZys S.A., San Carlos De Bariloche, Argentina) and the porphyrin Pt(II) octaethylporphyrin (PtOEP, >95%, Frontier Scientific, Logan, UT, USA) were used as received. Tetrahydrofuran (THF, HPLC grade, Cicarelli, Santa Fe, Argentina) was refluxed for 5 h with potassium hydroxide pellets (KOH, pro-analysis grade, Taurus, Córdoba, Argentina) and subsequently distilled. Ul-rapure water was obtained using Milli-Q^®^ Reference Water Purification System (Merck Millipore, Burlington, MA, USA).

Dulbecco’s modified Eagle’s medium (DMEM, Gibco^®^, Thermofisher Scientific, Waltham, MA, USA), Penicillin 10,000 units/mL—streptomycin 10,000 μg/mL solution Gibco, glutamine (GlutaMAXTM 100X Gibco R), sodium pyruvate 100 mM solution Gibco and phosphate buffered saline (PBS) were purchased from Life Technologies (New York, NY, USA).

### 2.2. Synthesis of Particles

For general experiments, F8BT (500 mg/L), PS-PEG-COOH (2000 mg/L), PtOEP (500 mg/L) and Fe_3_O_4_ or NiFe_2_O_4_ nanoparticles (5000 mg/L) were dissolved in distilled THF. Next, all solutions were mixed and diluted in THF varying the CP:IONPs mass ratio. The solutions were mixed to a final concentration of 50, 10 and 50 mg/L of F8BT, PS-PEG-COOH and IONPs (Fe_3_O_4_ or NiFe_2_O_4_) respectively for the 1:1 CP:IONPs mass ratio; 50, 10 and 320 mg/L of F8BT, PS-PEG-COOH and IONPs (Fe_3_O_4_ or NiFe_2_O_4_) respectively for the 1:6.4 CP:IONPs mass ratio; and 50, 10 and 640 mg/L of F8BT, PS-PEG-COOH and IONPs (Fe_3_O_4_ or NiFe_2_O_4_) respectively for the 1:12.8 CP:IONPs mass ratio. IONP-doped CPNs functionalized with PS-PEG-COOH in aqueous solution were prepared by the nanoprecipitation method [8,11], particles are labeled as CPFeNP for CPN doped with Fe_3_O_4_ IONPs and CPNiNP for CPN doped with NiFe_2_O_4_ IONPs. Briefly, 5 mL of the F8BT/PS-PEG-COOH/IONPs solution in THF was quickly added to 10 mL of H_2_O while sonicating (PS-30A Arcano, Buenos Aires, Argentina). Later, THF and H_2_O were removed under reduced pressure (using a rotary evaporator) yielding a final volume of 5 mL. Finally, the concentration of F8BT in the resulting dispersion was recalculated by comparing absorption spectra before and after injection in H_2_O. For MRI and fluorescence imaging experiments IONP-doped CPNs having a CP:IONPs mass ratio of 1:6.4 were used exclusively.

For PDT experiments, porphyrin-doped CPNs with or without IONPs (Fe_3_O_4_ or NiFe_2_O_4_) were prepared. Nanoparticles having a 1:6.4 CPN:IONPs mass ratio were chosen for evaluation of PDT protocols. Porphyrin-IONP-doped CPNs were prepared as follows, F8BT, PS-PEG-COOH, PtOEP and IONPs were mixed in THF to a final concentration of 50, 10, 5 and 320 mg/L, respectively, and then 5 mL of the mixture were injected in 10 mL of water under sonication. Afterwards, THF and water were removed under reduced pressure in a rotary evaporator to a final volume of 5 mL. Porphyrin-doped CPNs were prepared in the same manner but without the addition of IONPs. 

Unless otherwise noted, given particle concentrations are expressed in terms of F8BT mass concentration.

### 2.3. Steady State Absorption & Emission Techniques

UV-VIS absorption spectra were recorded on a diode-array spectrophotometer (Agilent Hewlett-Packard, HP 8452A, Agilent Technologies Inc., Santa Clara, CA, USA) in 1 cm quartz cuvettes at room temperature. Emission measurements were acquired from dilute solutions (Abs max < 0.1) in 1 cm cuvettes at room temperature and with excitation at the sample absorption maximum. Corrected emission spectra were recorded with a research spectrofluorometer (Fluoromax-4, Horiba, Kioto, Japan).

### 2.4. Dynamic Light Scattering

Size distribution evaluations of IONP-doped CPN suspensions in water were performed by dynamic light scattering (DLS) on a Zetasizer Nano ZS90 (Malvern Instruments, Malvern, Worcestershire, UK). The measurements were done on a cell type: ZEN0118-low volume disposable sizing cuvette, setting 2.420 as the refractive index with 173° backscatter (NIBS default) as the angle of detection. The duration of the analysis was set as automatic and three as the number of measures.

### 2.5. TEM Analysis

Transmission electron microscopy (TEM) images were obtained on an FEI Tecnai G2 Twin (Thermofischer, Waltham, MA, USA) microscope operated at an accelerating voltage of 100 kV. TEM samples were prepared by dropping a solution of the corresponding IONP-doped CPNs on a carbon-coated copper grid and letting the solvent evaporate.

### 2.6. Cell Culture

To perform the cytotoxicity and in vitro uptake analysis, adherent human GBM tumor cells U-87 MG (ATCC^®^ HTB-14™) and T98G (ATCC^®^ CRL-1690™) were cultured in an incubator under humidified atmosphere at 37 °C with 5% CO_2_ in DMEM supplemented with 10% *v/v* FBS and lifted by incubation in 0.25% trypsin/1 mM ethylene-diaminetetraacetic. To develop the animal models for in vivo studies, authenticated rat glioma C6 cells obtained from the American Type Culture Collection (number CCL-107, Manassas, VA, USA) were grown in DMEM supplemented with 10% fetal bovine serum, penicillin gentamicin and streptomycin.

### 2.7. Cytotoxicity and In Vitro Uptake Analysis 

To evaluate the toxicity of IONP-doped CPNs towards U-87 MG and T98G cells, MTT and Live/Dead cell viability assays were employed. For MTT assay, cells were seeded in 96-well plates (2 × 10^4^ cells/well) and exposed to increasing concentrations of CPFeNP or CPNiNP (0, 3, 6, 12, 24 and 48 mg/L) for 24 h and cell viability was evaluated using the MTT colorimetric assay as previously described [8]. For Live/Dead cell viability assay, cells were seeded in 24-well plates (1 × 10^5^ cell/well) and exposed to increasing concentrations of CPFeNP or CPNiNP (0, 3, 6, 12, 24 and 48 mg/L) for 24 h. Afterwards, cells were washed, lifted and incubated with LIVE/DEAD™ (Invitrogen, Molecular Probes, Carlsbad, CA, USA) Fixable Far Red Dead Cell Stain probe and analyzed by flow cytometry using a Guava easyCyte 6-2 L flow cytometer with excitation and emission at 642 nm and 661/15 nm, respectively. Cellular uptake of nanoparticles was analyzed at the same time with the flow cytometer taking advantage of the particle’s intrinsic fluorescence using the excitation and emission at 488 nm and 525/30 nm, respectively.

### 2.8. PDT In Vitro Treatment

U-87 MG cells (2 × 10^4^ cells/well) were seeded in 96-well plates and exposed to PtOEP-IONP-doped CPNs or PtOEP-doped CPNs (12 and 24 mg/L) for 24 h and, after that time, cells were washed with PBS to remove traces of not incorporated nanoparticles. Then, culture medium was replaced with fresh 10% FBS supplemented DMEM. Afterwards, cells were irradiated with a MultiLED system (460 ± 20 nm) with an irradiance (flux density) of 68 mW/cm^2^ for 10 min (equivalent to a dose of 40 J/cm^2^). Viability was examined 24 h after illumination using MTT assay as described previously and results were expressed as percentage relative to control cells (cells non-irradiated and without nanoparticles) [10].

### 2.9. Magnetic Resonance Imaging 

MRI studies were performed on a 7.0-Tesla Bruker Biospec^®^ system (Bruker Medical Gmbh, Ettlingen, Germany). A 40 mm transmitter coil with a 23 mm surface coil as a receiver was used for mouse brain imaging, whereas a selective birdcage resonator of 40 mm was employed to perform the heterotopic mouse model evaluation and the in vitro experiments with phantoms. MR images were acquired using ParaVision 6.0.1 software operating on a Linux environment. 

### 2.10. Fluorescence Imaging

In vitro and ex vivo fluorescence studies were carried out using an IVIS Lumina II ^®^ Optical Imaging Systems (Caliper, PerkinElmer, Inc., Walthman, MA, USA). Fluorescence images were analyzed using Aura software (Spectral Instruments Imaging^©^, Tucson, AZ, USA).

### 2.11. Imaging Phantom Studies

A phantom, including three different solutions (300 μL) of IONP-doped CPNs (1:6.4 CP:IONPs mass ratio), was prepared in an 8-well chamber (Ibidi GmbH, Martinsried, Germany). Concentrations employed—expressed as parts per million of F8BT—were: 5, 25 and 50 mg/L. Two additional wells, the corresponding nanoparticle (20 mg/L) without the magnetic center (CNP/no-met) and PBS, were used as controls. Besides, to determine the relaxivities *r*_2_ of the compounds, two additional phantoms using 200 μL Eppendorf were prepared containing 7 decreasing concentrations of IONP-doped CPNs: 25, 12.5, 6.25, 3.13, 1.56, 0.78 and 0.39 mg/L related to the polymer content.

T_2_-weighted images (T_2_WI) and T_2_ maps of the phantom were acquired in coronal orientation, employing fast sequences and the parameters indicated in Table 1. In mice, axial and/or coronal images were acquired with the same sequences.

The phantom was also studied by fluorescence IVIS Lumina right after MRI. A study was performed with the excitation and emission wavelengths corresponding to the nearly matching F8BT polymer absorption and emission maxima (460/540 nm) in order to select the adequate emission and excitation filters to study the IONP-doped CPNs. The settings were: autoexposure, F/stop = 2, medium binning and FOV = 7.5 × 7.5 cm^2^. 

### 2.12. Development of GBM In Vivo Models

The experimental procedures were approved by the highest institutional ethical committee (Community of Madrid) and met the national (R.D. 53/2013) and the European Community guidelines (2010/62/UE) for care and management of experimental animals. Animals were housed in the animal premises of our institution (Reg. No. ES280790000188) and cared by specialized personnel. Male and female NOD-SCID mice, 8–12 weeks, were employed to develop two tumor mouse models with the same tumoral cells: one orthotopic model generated by intracranial cell injection, another heterotopic by subcutaneous inoculation. To that, C6 glioma cells were cultured until reaching confluence, when were detached, resuspended in medium and counted for cell implantation.

For orthotopic implantation, mice were anesthetized in an induction box using 2–3% isoflurane/O_2_ at a flow of 0.8 L/min. Afterwards, they were placed in a stereotaxic device where anesthesia was maintained and delivered through a nose mask (1–1.5% isoflurane/O_2_). In order to prevent cornea from drying out, the eyes of the mice were covered with Vaseline. A midline incision was made on the skull with a scalpel and a burr hole was punctured 0.23 mm right to the Bregma with a 25G needle. Then, using a Hamilton syringe, 10^5^ C6 cells in 10 μL of DMEM with 30% Matrigel matrix were dropped to a depth of 0.33 mm in the right caudate nucleus. The syringe was carefully removed, the drilled hole sealed with bone wax and the scalp sutured. Animals received buprenorphine subcutaneously (s.c.) as analgesia before implantation and during the following 2 days (0.1 mg/kg). 

The heterotopic inoculation was performed in anesthetized animals (1–1.5% isoflurane/O_2_) by injecting s.c. 10^5^ C6 cells in 100 μL of DMEM with 50% Matrigel into the two bilateral dorsal flanks of mice. Tumor diameters were measured once a week using a caliper.

In all cases, ortho- and heterotopic glioma bearing animals were supervised every 2–3 days. 

### 2.13. Preclinical MRI Studies

Animals were anesthetized in an induction box before placing in the MRI equipment, where the anesthesia was supplied through a nose mask. The body temperature was maintained at 37 °C using a heating blanket and respiratory rate was monitored during all the experiments with a pneumatic pillow placed under the thorax. Images were acquired employing fast spin-echo sequences in coronal orientation for body studies and in axial orientation for brain and flank tumor evaluation.

Biodistribution (pharmacodynamics) and tumor evaluation (T_2_WI and T_2_ maps) studies used the following protocol and the acquisition parameters indicated in Table 1:

1. T_2_WI and T_2_ map acquisitions.
2. Dynamic T_2_WI study in which 200 μL of IONP-doped CPN 1:6.4 solutions (50mg/L and corresponding to 320 mg/L of IONPs) flushed with 100 μL of saline, were injected intravenously after acquiring 4 basal images.
3. T_2_WI and T_2_ map 30 min after the CPN injection.

#### 2.13.1. IONP-Doped CPN MRI Evaluation of Orthotopic Tumors

Glioma brain-bearing mice (*n* = 12) were studied when the tumor reached an advanced stage (approx. volume 30–50 mm^3^). At that point, T_2_W images, T_2_ maps and dynamic studies were performed with the acquisition parameters specified in Table 1 (mouse-head). To assess the biodistribution and tumor accumulation of the nanoparticles, tumor and apparently healthy regions in the contralateral hemisphere were studied. Two mice were also submitted to T_2_WI and T_2_ map acquisitions at 0.5, 1 and 3 h after the nanoparticle administration.

Two additional animals were employed for nanoparticle intratumor (i.t.) administration. In that case, 10 μL of IONP-doped CPNs 1:6.4 (containing 25 mg/L F8BT and 160 mg/L IONPs) were directly injected in the tumor by placing the animal in the stereotaxic device after localizing the cerebral coordinates in T_2_W images. In that case, T2WI were acquired before and after the i.t. injection. 

#### 2.13.2. MRI Evaluation of Heterotopic Tumors

When tumors in flank reached a pre-determined volume (approx. 100–150 mm^3^), the same MRI protocol as the one employed in the orthotopic model was carried out but using the parameters indicated in Table 1 (mouse-body, *n* = 10). To assess longer periods of time, two mice were T_2_WI and T_2_ map tested at 1, 3, 6 and 24 h after the nanoparticle administration. The biodistribution of the nanoparticles in the heterotopic mouse model was tested in the following organs: liver, kidney and tumor.

Furthermore, additional mice (*n* = 2) were submitted to nanoparticle intratumor injections in both flanks (50 μL, containing 50 mg/L F8BT and 320 mg/L IONPs). In a similar procedure as the intracranial glioma, T_2_WI were obtained before and after the i.t. CPNs administration.

#### 2.13.3. Image Processing and Analysis

T_2_W images were analyzed without any additional treatment. T_2_ maps were generated, with the MyMapAnalyzer home-made software develop in MatLab (MathWorks, Nattick, MA, USA), on a pixel-by-pixel base by adjusting the signal intensity to Equation (1):

(1) $$S_{\left(TE\right)}=S_{0}\;e^{-TE/T_{2}}$$

where *S*_(*TE*)_ is the signal intensity of a pixel in an image at a specific TE value, and *S*_0_ is the expected data for TE = 0, and TE the different values employed in the acquisition.

T_2_ maps from IONP-doped CPNs at different concentrations in phantom were used to calculate relaxivity of the compounds, obtained with Equation (2).

(2) 1T2(obs)=1T2(solv)+r2[CPN]

where *T*_2_(*obs*) is the relaxation time measured at each value of concentration, *T*_2_(*solv*) is the T_2_ value of the medium without the presence of nanoparticle, and [CPN] is the concentration of IONP-doped CPNs employed expressed as mg/mL of F8BT.

To analyze the biodistribution studies, 3 regions of interest (ROIs) from different MRI slices were manually placed in the liver, renal cortex, renal medulla and tumors in the heterotopic glioma model. In the intracranial glioma, three ROIs of the same size were manually placed in the tumor and three in the contralateral hemisphere to be used as healthy brain tissue. Then, the signal intensity from images was evaluated along time. The same ROIs selection procedure was carried out for evaluating particle accumulation in the analysis of T_2_ maps for the orthotopic model. 

### 2.14. Statistical Analysis

Data were analyzed using GraphPad Prism Software, version 8.412 (GraphPad Software, La Jolla, CA, USA), and presented as mean ± standard error of the mean (SEM). Brown-Forsythe and Welch ANOVA tests with multiple comparations using Dunnett T3 post hoc analyses were performed. P values lower than 0.05 were considered to be statistically significant. 

### 2.15. Euthanasia and Organ Preservation for Fluorescence Imaging

Mice were euthanized by cervical dislocation at different time points after IONP-doped CPN i.v. injection (30 min, 1 h, 3 h, 6 h or 24 h) and at 1 h after PBS administration as control group. Vital organs such as liver, kidneys, spleen and flank tumors were harvested in 4% paraformaldehyde for 24 h and then preserved in PBS until further studies.

### 2.16. Ex vivo Fluorescence Studies

Organs preserved in PBS were studied by fluorescence using the following parameters: 460/540 nm excitation/emission filters, exposure = 40 s, F/Stop = 2, Medium = 4 and FOV = 12.5 × 12.5 cm^2^. To analyze fluorescence images, 2 ROIs per organ were selected and mean fluorescence efficiency of each ROI was quantified.

The whole experimental setup followed in the presented work is schematically depicted in Figure S1.

## 3. Results

### 3.1. Preparation and Characterizations of IONP-Doped CPNs

The hydrophobic interactions between F8BT chains, polystyrene backbones and oleic acid capped IONPs produced densely packed IONP-doped CPNs. The IONPs, F8BT conjugated polymer, and PS-PEG-COOH were all dissolved in THF, and this solution was quickly injected into water under sonication to allow the nanoparticles’ formation. Considering the excellent solubility of polyethylene oxide in water, most PEG-COOH side chains are expected to be extended towards the aqueous solution providing colloidal stability. Given their small size, ~5 nm, the used IONPs can be considered superparamagnetic [29]. This property presumably improves the colloidal stability of the resulting nanoparticles and plays a critical role in achieving good image contrast in MRI [30,31].

**Figure 1:**
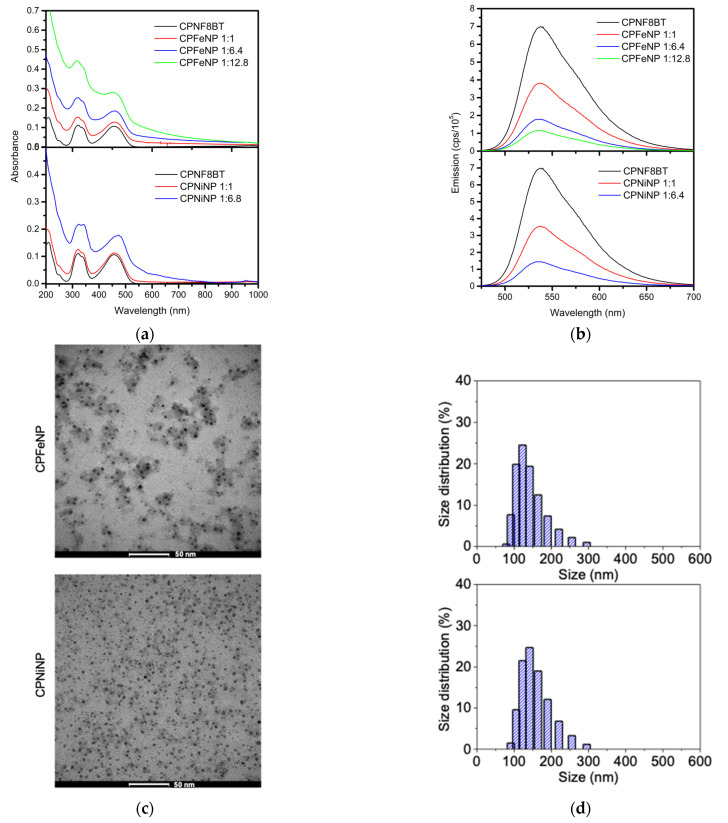
Characterization of IONP-doped CPNs. (**a**) Absorption spectra of CPFeNP (upper panel) and CPNiNP (lower panel) at different CP:IONPs mass ratios in water. (**b**) Emission spectra of CPFeNP (upper) and CPNiNP (lower) at different CP:IONPs mass ratios in water.(**c**) Representative TEM images of CPFeNP (upper) and CPNiNP (lower). (**d**) Size distribution histograms of CPFeNP (upper) and CPNiNP (lower).

In order to determinate the optimal conditions for MRI and in vivo fluorescence experiments, the ratios of F8BT and IONPs were varied obtaining IONP-doped CPNs dispersions in water with different degrees of turbidity and colloidal stability. Higher concentration of IONPs resulted in greater turbidity and lower colloidal stability of the resulting particles. Partial precipitation was observed for particles having a 1:12.8 CPN:IONPs mass ratio. Absorption and emission spectra were recorded for the IONP-doped CPNs. It was found that at high concentration of IONPs (both Fe_3_O_4_ and NiFe_2_O_4_) the absorption spectra of the resulting particles (CPFeNP and CPNiNP) show significant contribution of the IONPs and presumably some light scattering (Figure 1). Nevertheless, the characteristic absorption peaks of F8BT are observed in all the spectra, indicating that the CP was incorporated into the IONP-doped particles and its main absorption transitions were not significantly affected by the presence of IONPs. On a different note, the emission spectra of all IONP-doped CPNs showed weaker emission as compared to CPNs without IONPs centers and this was more prominent in the IONP-doped CPNs with the higher CP:IONPs ratios. These results indicate that IONPs introduce an additional route (or accelerate a previous one) for the deactivation of F8BT singlet excited state. This quenching effect is typically seen in fluorescent magnetic nanoparticles [23].

Based on these results, MRI and fluorescence imaging experiments were performed using IONP-doped CPNs with the CP:IONPs ratio of 1:6.8. 

The morphology of the synthesized CPFeNP and CPNiNP nanoparticles was investigated by TEM. Representative TEM images displayed in Figure 1c, show that particles have a relatively uniform spherical shape. Additionally, it is possible to observe that almost all of the IONPs (darker centers, consistent with the presence of heavier atoms, Fe or Ni) are embedded within the larger polymeric organic nanoparticles (less dense layer associated with the presence of mostly C and H atoms). A few agglomerates can also be seen in the TEM image of as deposited nanoparticles. Particle size in solution was measuring using DLS. The mean particle diameters of the CPFeNP and CPNiNP in water were 140 ± 9 nm and 146 ± 19 nm respectively (Figure 1d). These mean diameters are smaller than those previously reported for similar IONP-doped CPNs [23,26]. The relatively small size and narrow distribution of our IONP-doped CPNs makes them attractive candidates to be used as bimodal probes for in vivo fluorescence/magnetic resonance imaging.

### 3.2. Biocompatibility and Nanoparticle Uptake in GBM Cells 

To evaluate the inherent cytotoxicity of IONP-doped CPNs, two GBM cell lines (U-87 MG and T98G) were exposed to increasing CPFeNP or CPNiNP concentrations (3.75–60 mg/L) for 24 h in analogy with previous studies from our group [8,10]. Then, MTT and LIVE/DEAD cell viability assays were performed to determinate mitochondrial reducing activity and cell membrane integrity, respectively. Viability results for both GBM cell lines are shown in Figure S2. Cells incubated with either CPFeNP or CPNiNP (3.75–30 mg/L) for 24 h did not show statistically significant differences with respect to the control group (0 mg/L) resulting in viability percentages superior to 90%. Only the highest concentration tested (60 mg/L) showed a small but statistically significant reduction in cell viability for both GBM cell lines with a higher cytotoxic effect in U-87 MG cells. Furthermore, cell morphology in bright-field images was not significantly affected upon incubation with IONP-doped CPNs (not shown).

**Figure 2:**
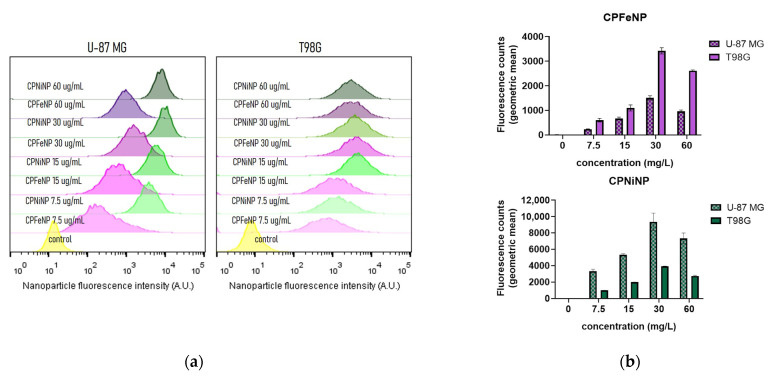
IONP-doped CPN cell uptake quantification by flow cytometry. (**a**) Cell fluorescence histograms obtained from U-87 MG and T98G GBM cell lines after 24 h nanoparticle incubation time. (**b**) Bar graphs representing semi quantification (geometric mean fluorescence intensity) of cell fluorescence (associated with particle uptake) at varying particle concentrations for CPFeNP (upper panel) and CPNiNP (lower panel) respectively.

Taking advantage of the particle’s intrinsic fluorescence, IONP-doped CPN cell incorporation was studied using flow cytometry. As shown in steady state fluorescence emission experiments (Figure 1b), IONPs produced significant fluorescence quenching (~80%) of CPNs at the 1:6.8 CPN:IONP mass ratio. However, given the exceptionally large absorption cross section and high emission quantum yield of F8BT we speculated that the remaining fluorescence emission intensity of our IONP-doped CPNs was sufficiently high for the detection of particles by flow cytometry. Figure 2 displays flow cytometry cell fluorescence histograms and a semi quantitative cell fluorescence comparison between U-87 MG and T98G cell lines exposed to increasing IONP-doped CPN concentrations. Cell fluorescence intensity increase above the control value is associated with nanoparticle uptake by cells. Both cell lines incorporated CPFeNP and CPNiNP in a concentration dependent manner up to 30 mg/L, and the cell uptake was superior in U87 MG cells. At 60 mg/L, a decrease in mean fluorescence values in both cell lines was observed, this decrease is likely related to the reduction in cell viability observed at this concentration.

### 3.3. PDT In Vitro Efficacy of IONP-Doped CPNs 

**Figure 3:**
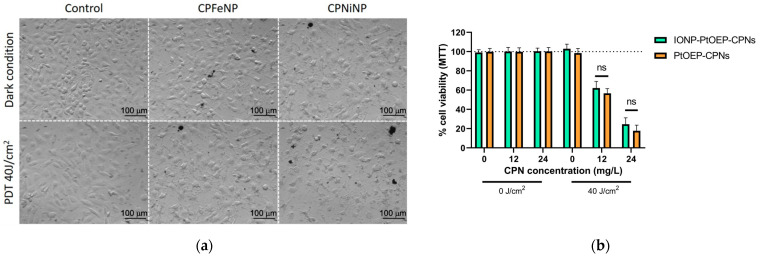
Photodynamic therapy of GBM in vitro using PtoEP-doped CPNs with and without IONPs. (**a**) Brightfield images of GBM cell lines before (top line) and after (bottom line) light irradiation (460 nm, 40 J/cm^2^) of PtOEP-IONPs-doped CPN (CPFeNP and CPNiNP). Control experiment in the absence of nanoparticles is also shown for comparison. (**b**) U-87 MG cell viability percentages quantified by MTT assay 24 h after PDT (see details in experimental section) using IONP-PtOEP-CPNs (green bars) and PtOEP-CPNs (orange bars) (ns = no statistically significant differences).

In order to evaluate if metallated porphyrin-doped CPNs conserved photodynamic activity after the doping with IONPs, PtOEP-IONP-doped CPNs were prepared and their PDT activity was evaluated and compared with that of PtOEP-doped CPNs. Figure 3 shows the viability of U-87 MG cells incubated with two concentrations of nanoparticles (12 and 24 mg/L) and irradiated with 40 J/cm^2^ light doses. A significant toxic effect was found using either PtOEP-doped CPNs or IONP-doped CPNs after blue light irradiation (Figure 3b). The resulting viability values were similar to previous reports from our group [8,10]. No statically difference was found between PtOEP-doped-CPNs and IONP-PtOEP-CPNs suggesting that the incorporation of IONPs into CPNs does not significantly affect the generation of ^1^O_2_ and subsequent oxidative damage to cells. After PDT, cells clearly showed changes in cell phenotype (apoptotic appearance), cell detachment and debris in both nanoparticle treatments (Figure 3a).

### 3.4. In Vitro Imaging Phantom Studies

Comparative results obtained from the T_2_ maps of a phantom containing both types of IONP-doped CPNs at three different concentrations are shown in Table 2.

Relaxivities *r*_2_ determined from T_2_ maps of the corresponding phantoms (Figure S3) were 3.80 ± 0.04 for CPFeNP and 2.30 ± 0.02 s^−1^ [F8BT]^−1^ for CPNiNP.

Fluorescence studies showed a slightly higher intensity signal in CPNiNP solutions than in CPFeNP solutions. Fluorescence intensity decrease linearly with decreasing particle concentrations (not shown). The fluorescence and MRI measurements were repeated a few times after several days obtaining similar *r*_2_ and fluorescence values. This confirms that IONPs-CPNs are stable under the studied conditions.

### 3.5. Preclinical MRI Studies Subsection

#### 3.5.1. Biodistribution

**Figure 4:**
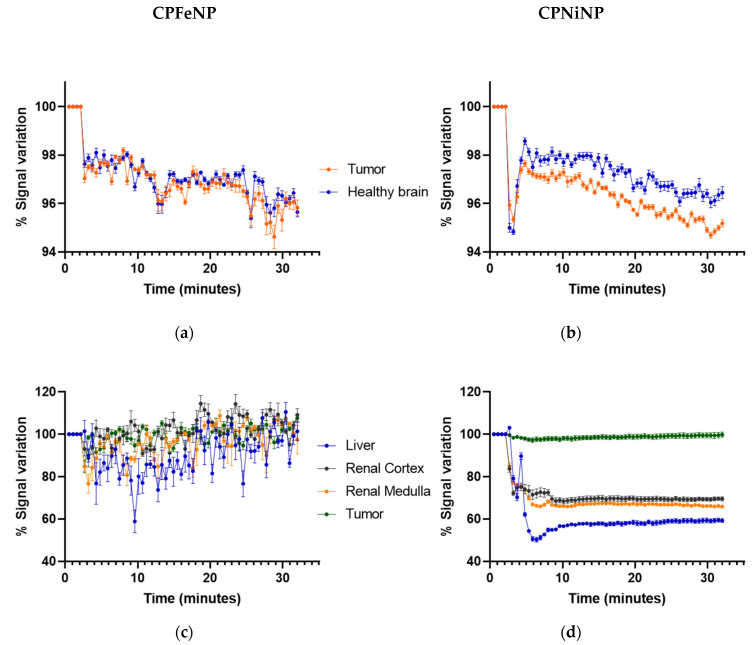
Biodistribution MRI studies of ION-doped CPNs. Graphics show the variation in MRI signal intensity (mean ± SEM) in the different organs evaluated: tumor and contralateral healthy brain in the orthotopic glioma model for CPFeNP (**a**) and CPNiNP (**b**); and liver, renal cortex, renal medulla and tumor in the heterotopic GBM model for CPFeNP (**c**) and CPNiNP (**d**).

Time-resolved biodistribution results from dynamic T_2_W MRI acquisitions for both IONP-doped CPNs are shown in Figure 4 for studies of glioma bearing mice, either orthotopically (Figure 4a,b) or heterotopically (Figure 4c,d).

The CPNiNP showed clearly a higher effect in the tumor than in the contralateral healthy brain, pointing to a major accumulation in the former tissue. Regarding to the body biodistribution, CPNiNP preferentially accumulated in kidneys and liver, while almost no effect was appreciated in the other assessed tissues. On the contrary, CPFeNP induced the same effects in the healthy brain and orthotopic tumors, and essentially identical effects in almost all studied organs, except for a slightly higher accumulation in liver. 

#### 3.5.2. Evaluation of the Orthotopic Glioma Model

**Figure 5:**
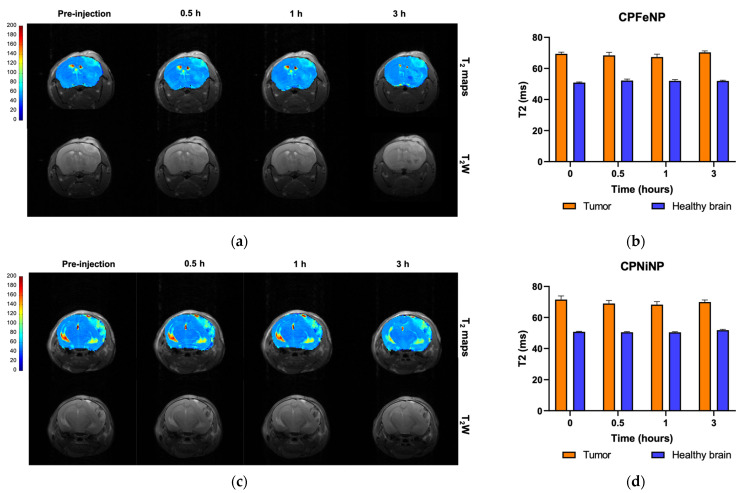
T_2_ color-code based maps and T_2_W images of orthotopic GBM model. Data were acquired before the i.v. administration of the IONP-doped CPNs, and at 0.5, 1 and 3 h after the injection. MRI evaluation of CPFeNP (**a**) and CPNiNP (**c**). Graphical bar showing the variation in T_2_ values (mean ± SEM) along the temporal evaluation of CPFeNP (**b**) and CPNiNP (**d**).

Representative T_2_W images and T_2_ maps of glioblastoma bearing mice, and comparison of absolute T_2_ values are shown in Figure 5 for both nanoparticles along the temporal evolution. 

Comparison of quantitative data from these studies are also presented in Figure 5b,d The tumor presented higher T_2_ values due to the increased vascularity and edema/inflammation processes, but neither the tumor nor the contralateral brain experienced a statistically significant decrease in T_2_ with time after i.v. administration of nanoparticles. 

**Figure 6:**
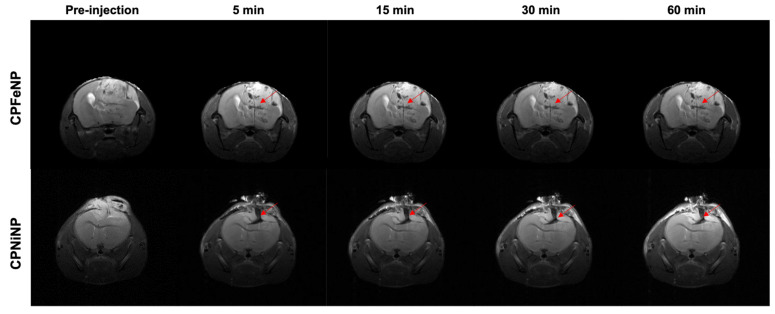
MRI monitoring after i.t. injection of IONP-doped CPNs in the brain. T_2_W axial images of the brain were acquired at 5, 15, 30 and 60 min after the i.t. nanoparticles injection. Red arrows point to the tumor region where the nanoparticles can be identified as a hypointense signal.

Figure 6 shows the images obtained from intratumoral injection of IONP-doped CPNs, where nanoparticle accumulation is appreciated as a dark or hypointense area due to the presence of the metal.

#### 3.5.3. Evaluation of the Heterotopic Glioma Model

**Figure 7:**
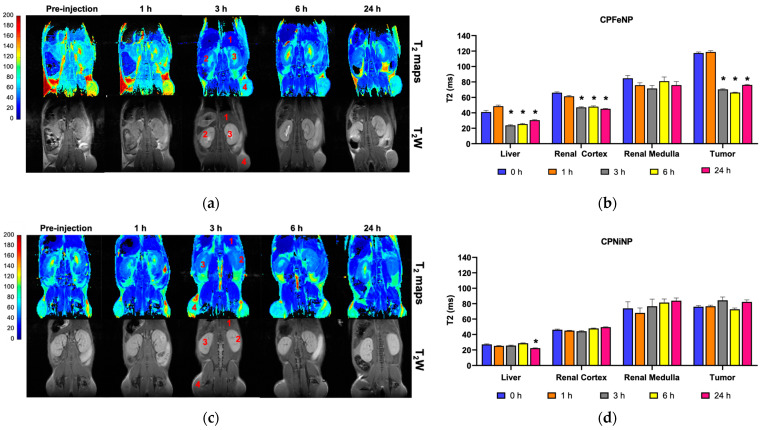
T_2_ color-code based maps and T_2_W images of heterotopic GBM model. T_2_ color-code based maps and T_2_W images acquired before the i.v. administration of the IONP-doped CPNs, and at 1, 3, 6 and 24 h after the injection. Numbers indicated the tissues where the ROIs were selected to do the measurements: 1, liver; 2, renal cortex; 3, renal medulla; 4, tumor. (**a**) CPFeNP MRI evaluation; (**c**) CPNiNP MRI evaluation. Graphics show the variation in T_2_ values (mean ± SEM) along the temporal evaluation: (**b**) CPFeNP; (**d**) CPNiNP. Statistical symbols correspond to the comparison of the data to the pre-injection value (* *p* < 0.05).

Results from equivalent studies to the previous section but with heterotopic glioma mice are presented in Figure 7. T_2_W images and T_2_ maps correspond to one mouse for each IONP-doped CPN administration.

Data showed that CPFeNP induced a significant decrease in T_2_ values at long times (3 h and beyond) after nanoparticle administration in all tissues assessed except for the renal medulla, while significant effects of CPNiNP were detected only in liver 24 h post-injection.

**Figure 8:**
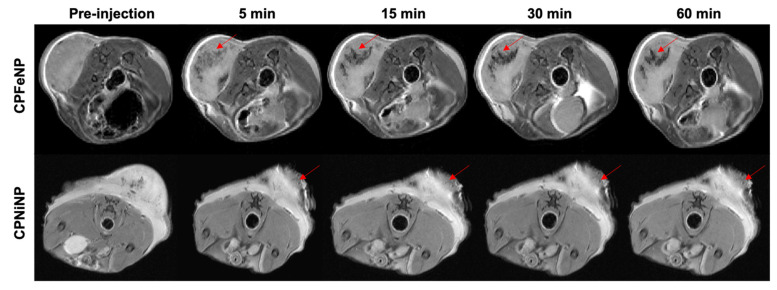
MRI monitoring after i.t. injection of IONP-doped CPNs in the flank. T_2_W axial images of the mouse body were acquired at 5, 15, 30 and 60 min after the i.t. nanoparticles injection. Red arrows point to the tumor region where injected nanoparticles (CPFeNP: upper panels, CPNiNP: lower panels) can be identified as a hypointense signal.

In Figure 8, MR images obtained after i.t. injection of IONP-doped CPNs are presented for two mice as example. The region where particles accumulate can be clearly identified as a dark signal in the images. It was also noted the non-diffusivity of the nanoparticles solution after being injected in the tumor, the hypointense signals did not significantly change with time neither in shape nor in position. 

### 3.6. Fluorescence Ex Vivo Studies

**Figure 9:**
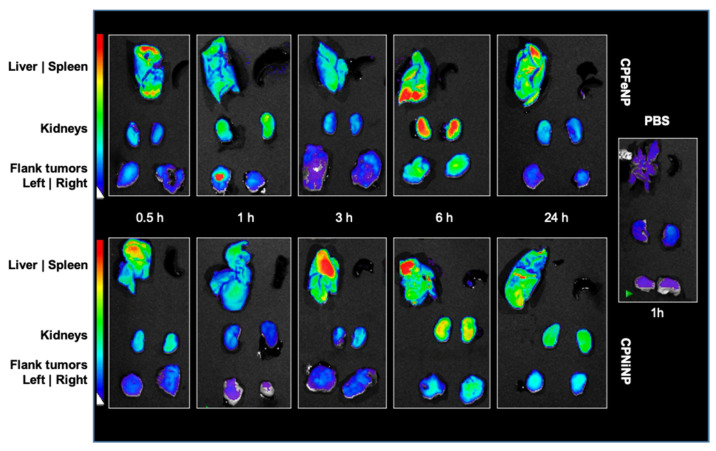
Fluorescence images of organs from mice euthanized at different times after IONP-doped CPN administration. Fluorescence images of liver, spleen, kidneys and flank tumors at different time points (20.5, 1, 3, 6 and 24 h) after i.v. injection of CPFeNP (upper panels) and CPNiNP (lower panels). Control: PBS (1 h) image corresponds to organs from a mouse sacrificed 1 h after the injection of PBS.

Fluorescence images were acquired for organs excised at several time points after i.v. administration of CPFeNP and CPNiNP solutions. Organ images of mice euthanized at 1 h after the i.v. injection of PBS, were also obtained to be used as controls. Figure 9 shows representative images as examples. 

Fluorescence imaging results indicate a higher accumulation of both CPNiNP and CPFeNP, in liver and kidneys as compared to heterotopic tumors in the flank. It is worthy to note that no fluorescence signal was detected in spleen for any of the assessed particles. In the control studies with PBS, very low fluorescence signals were appreciated.

Finally, to check if the nanoparticles were able to emit fluoresce within a tumor regardless of their ability to reach this tissue via i.v. administration, some animals were i.t. injected with solutions either of CPNiNP or CPFeNP (Figure S4). In this case, tumors injected with both IONP-doped CPNs showed more intense emission signals compared to the non-injected tumor. Additionally, it was observed that the intensity of overall fluorescence signal in the tumor of the mouse injected with CPNiNP was higher than that of the mouse injected with CPFeNP.

## 4. Discussion

The design and production of new nanostructured materials optimized for theranostic applications is a difficult task that requires careful choice of individual components, tuning of components proportion and overall size to achieve the desired therapeutic and diagnostic properties. In particular, the development of theranostic nanoparticles enabling in vivo monitoring using dual contrast strategies is highly desirable as it allows for redundant biodistribution analysis, and application of metronomically optimized treatment strategies based on such information. In this line, CPNs are suitable nanoplatforms to incorporate different dopants (organic and metallic) enabling multiple functions. Besides, the introduction of rare biological metals such as Ni (IONPs) or Pt (PtOEP) gives us the opportunity to quantify the CPN content in tissues using more sensitive techniques like ICP-MS in the near future [32]. Our groups have previously developed metallated porphyrin-doped CPNs with outstanding performance in PDT for the eradication of GBM tumor cells in vitro [8,9,10]. With the aim of a potential clinical therapeutic application of this type of particles, their preclinical biodistribution evaluation in animal models is mandatory particularly considering that pharmacokinetics and tumor accumulation information for these materials is extremely limited. To date, only a few articles have reported the incorporation of metallic centers into CPNs with the aim of adding contrast enhancement in MRI studies. Even fewer reports have evaluated this type of particles in vitro and in vivo in tumor bearing mice [23,26], however an exhaustive evaluation in relevant preclinical GBM models was not conducted to the present.

In the present study, we synthetized CPNs incorporating IONPs capped with oleic acid. Two types of commercial IONPs Fe_3_O_4_ and NiFe_2_O_4_ were chosen due to the excellent T_1_ and T_2_ relaxivities [33,34] with the aim to compare relaxation rates for MRI studies and the interaction or interference with fluorescent emission and singlet oxygen production of the resulting particles. Adding IONPs into CPNs resulted in an increment of mean size and broadening of the size distribution as compared to CNP/no-met [8]. Additionally, depending on the used CP:IONPs mass ratio, the colloidal stability and light emission intensity of the resulting nanoparticles were compromised. Based on these results, the CP:IONPs mass ratio of 1:6.8 was chosen as optimal for dual MRI and fluorescence imaging evaluation. Imaging studies relied in the acquisition of T_2_W images to qualitatively localize the CPNs and T_2_ maps to allow quantifying the effect on the MRI signal, proportionally related to the nanoparticle accumulation.

Both IONP-doped CPNs were biocompatible with human GBM cell lines at concentrations up to 30 mg/L after incubation for 24 h. Furthermore, PtOEP-IONP-doped CPNs were capable of inducing cell death upon light irradiation consistent with previous reports from our group using analogous PtOEP-CPNs [8,10]. Intracellular accumulation of IONP-doped CPNs was shown to be dependent on both cell type and particle type (Figure 2). Superior uptake was observed for CPNiNP in U-87 MG cells. This higher uptake is likely correlated with the observation of cytotoxicity at lower particle concentration (of the solution used for incubation).

In addition to providing good MRI contrast, our developed particles having tetrapyrrole, IONP-PtOEP-doped CPN, were shown to perform as efficient materials for PDT of glioma cell lines in vitro thus demonstrating their theranostic potential for the treatment of GBM [2,35,36]. Further experiments aimed to assess the mechanism and efficiencies of singlet oxygen generation of IONP-CPNs are needed to confirm cellular in vitro findings. It is reasonable to suppose that these mechanisms and efficiencies are similar to those of porphyrin-doped CPNs having identical photoactive components and previously reported from our group [8,10]. The superior PDT effect observed of CPNiNP in U-87 MG cells (Figure 3a) could be also in principle be attributed to the increased nanoparticle uptake for this type of particles and cell line.

The behavior of both IONP-doped CPNs as potential dual imaging contrast agents, was evaluated in vitro, ex vivo and in vivo, with fluorescence imaging and MRI. In vivo studies were performed in a C6 glioma bearing mice rather than in humanized glioma models because C6 cells can effectively simulate: the high growth rate, the high vascularization, and the highly infiltrative character of human GBM [37]. Being similar the behavior of both CPNs in the two imaging techniques, CPNiNP presented slightly better contrast features in fluorescence and MRI studies as compared to CPFeNP. Malignant gliomas possess a combination of an intact blood–brain barrier (BBB) and also a blood–brain tumor barrier (BBTB) which is formed during tumor progression [2]. Both barriers represent the major impediment structures to reach tumor tissue for most of the i.v. administrated nanoparticles [38,39]. Is well known that nanoparticle size affects their permeation from blood vessels into brain tumor tissue [40]. In our studies in the GBM orthotopic model, we appreciate a decrease in the MRI signal intensity of T_2_W images with both nanoparticles (Figure 4a,b), that is maintained at least 30 min after i.v. injection. While CPFeNP depicted a similar accumulation pattern in tumor and contralateral parenchyma, CPNiNP showed a differential contrast in both regions, indicating a higher uptake in glioma probably due to the enhanced permeability and retention (EPR) effect. This phenomenon is consistent with the previously mentioned superior cell uptake evidenced from in vitro studies. The size of our CPNs can be hindering a higher effect on T_2_WI signals and T_2_ values. Previous works reported a higher contrast by using nanoparticles and GBM mouse models [41,42,43], although in those studies their particles had smaller hydrodynamic diameters (<100 nm) than ours (130–140 nm). In fact, nanoparticles smaller than 100 nm are more likely to cross the tumor disrupted BTB and accumulate by EPR effect [44,45]. Although particle aggregation was evaluated in water, we certainly do not think that the existence of aggregation process in vivo could be limiting the GBM uptake. The inclusion of an amphiphilic copolymer stabilizer (PS-PEG-COOH) into IONP-doped-CPNs allows achieving high colloidal stability into physiological media. Previous works by our group have shown that CPNs suspended in growth medium supplemented with FBS show excellent colloidal stability [8]. Considering their analogous composition, it is reasonable to assume that the new nanoparticles have similar colloidal stability in physiological media. To compare the in vivo T_2_ enhancement capacity of our compounds with commercial nanoparticles contrast agents, we performed similar experiments by using Endorem^®^ (20 μmol Fe/kg body weight) in both orthotopic and heterotopic GBM animals. The results in the studies were very similar to those obtained with our CNPs in all the evaluated organs, although with a slightly lower effect on the healthy brain and the orthotopic tumor (Figures S5 and S6).

On the other hand, when the IONP-doped CPNs were injected intracranially into the tumor zone, nanoparticle accumulation is appreciated as a dark or hypointense area further demonstrating the ability of CPNs to act as contrast agents. Taking consideration of the amenable surface-functionalization of CPNs to target specific cells, bioconjugation with ligands, aptamers, peptides or antibodies towards BBB receptors represent the next challenge to mediate transcytosis and overcome these barriers to penetrate the brain and finally reach the tumor cells [46,47,48]. It is important to highlight here that there is only one site where the nervous system of mammals is directly exposed to the external environment. This is the roof of the nasal cavity which represents the intranasal route and has been investigated for nanoparticle entry into the brain bypassing the BBB [49]. Direct uptake of nanoparticles into the nervous system might occur in this site thus it can be another viable administration route that deserves to be explored.

On a different note, the evaluation of biodistribution and tumor accumulation using GBM heterotopic model revealed a decreased in MRI signal intensity and an increment in fluorescent signal in flank tumors associated with IONP-doped CPN accumulation. MRI studies with this heterotopic glioma model showed that these nanoparticles required several hours to accumulate in tumors (Figure 7), in agreement with others compounds that reported 5–6 h to achieve accumulation [26] or even 48 h [43]. These results suggest that in this animal model, where the BBB did not interfere with particle permeation, a significant intratumor accumulation of IONP-doped CPNs is achieved. Also, the fluorescence images allowed to follow the nanoparticle accumulation in different organs by another imaging technique. Results in these studies agree with MRI data, showing the higher IONP-doped CPN accumulation 6 h after the administration. The experiments also confirmed that the nanoparticles experienced higher uptake in the liver, but not in the spleen, as it was also described in previous studies with similar nanoparticles [26]. 

We can conclude that the incorporation of IONPs into CPNs gives us a functionalization of our CPNs to evaluate the routes and destinations of nanoparticles after i.v. injection in relevant GBM models and with more sensitive spatial resolution techniques such as MRI. Nevertheless, this work suffers from some limitations. The obtained results, which are closely related to the size of the nanoparticles and the type of magnetic center used, provide us with information for subsequent in vivo PDT protocols and the need for a toxicological evaluation of the nanoparticle accumulation in liver and kidney and if there is a possible way of elimination or clearance by these routes. The lack of this information is clearly a handicap of this study once to investigate the elimination pathway of these materials is a crucial point for the translation to the clinical practice. Besides, although the in vivo validation of PDT therapy in GBM models would add robustness to the work, these experiments could not be done for unsolvable reasons, and we plan to do them in the future. A few reports have demonstrated the efficacy of in vivo PDT using blue light [50,51], which encourages us to continue investigating in the near future the therapeutic efficacy in vivo of these nanoparticles with blue light irradiation. More importantly, light delivery unto brain tissue using sophisticated fiber optics has been proven as a viable strategy to achieve efficient PDT regimens for GBM management.

## Figures and Tables

**Table 1:** Acquisition parameters employed in the MRI studies.

Object	Type	TR ^1^ (ms)	TE ^2^ (ms)	Av ^3^	FOV ^4^ (mm^2^)	Acq ^5^	Resolution (μm^3^)	Additional Parameters
Phantom ^6^	T_2_WI	3000	23	3	40 × 40	3 min 36 s	156 × 156 × 1000	RARE ^8^ = 8
	T_2_ maps	5000	12	1	40 × 40	10 min 40 s	312 × 312 × 1000	NE ^9^ = 50, RARE = 8
Mouse-body ^6^	T_2_WI ^11,12^	1000	17	3	40 × 40	2 min 27 s	156 × 156 × 1000	RARE = 4
	T_2_ maps ^11^	3000	7	1	40 × 40	6 min 42 s	312 × 312 × 1000	NE = 32, RARE = 8
	Dynamic ^11^	1000	16	1	40 × 40	32 min	312 × 312 × 1000	NR ^10^ = 60, RARE = 4
Mouse-head ^7^	T_2_WI ^12^	2500	26	4	23 × 23	4 min	90 × 90 × 1000	RARE = 8
	T_2_ maps ^12^	3000	7	1	23 × 23	6 min 42 s	180 × 180 × 1000	NE = 32, RARE = 8
	Dynamic ^12^	1000	16	1	23 × 23	32 min	180 × 180 × 1000	NR ^10^ = 60, RARE = 4

^1^ TR: repetition time; ^2^ TE: echo time; ^3^ Av: averages; ^4^ FOV: field of view; ^5^ Acq: acquisition time; ^6^ studies performed with the 40 mm birdcage resonator; ^7^ studies performed with the 23 mm surface coil; ^8^ RARE: acceleration factor; ^9^ NE: number of echoes; ^10^ NR: number of repetitions; ^11^ Acquisitions performed in coronal orientation, ^12^ Acquisitions performed in axial orientation.

**Table 2:** T_2_ values determined from T_2_ maps of IONP-doped CPNs at different concentrations.

Compound	Concentration (mg/L) ^1^	T_2_ (ms)
CPFeNP	50	37.2 ± 0.8
	25	84.3 ± 0.5
	5	237 ± 2
CPNiNP	50	33.1 ± 0.5
	25	106 ± 2
	5	200 ± 2
CPN/no-met ^2^	20	488 ± 7
PBS	-	508 ± 7

^1^ Concentration expressed in terms of F8BT mass content; ^2^ CPNs/no-met: nanoparticle without IONPs. IONP-doped CPNs have a CP:IONPs mass ratio of 1:6.8, e.g., for CP concentration = 50 mg/L IONPs concentration = 320 mg/L.

## Data Availability

The data presented in this study are available on request from the corresponding author due to the need for a formal data sharing agreement.

## Supplementary Materials

The following are available online at https://www.mdpi.com/article/10.3390/pharmaceutics13081258/s1, Figure S1: Overview of the experimental workflow followed in the preparation, characterization, in vitro and in vivo evaluation of the IONP-doped CPNs. The scheme shows the steps to synthesize and purified the IONP-doped-CNPs in the upper panels and the preclinical assessment of the nanoparticles in the lower, Figure S2: Biocompatibility evaluation of IONP-doped CPNs in GBM human cell lines. MTT viability quantification for (a) U-87 MG and (b) T98G. Cell viability quantified by flow cytometry using LIVE/DEAD cell viability assays for (c) U-87 MG and (d) T98G. (* *p* < 0.05), Figure S3: Relaxivities determination of IONP-doped CPNs. T_2_ maps of nanoparticles at decreasing concentrations: (a) CPFeNP and (b) CPNiNP. Graphical linear regression of relaxation rates (1/T_2_) versus increasing nanoparticle concentration: (c) CPFeNP and (d) CPNiNP, Figure S4: Fluorescence images of tumors after i.t. IONP-doped CPN administration. Images show excised tumors from a control mouse without particle administration (left), a flank tumor after CPFeNP i.t. injection (middle) and a tumor after CPNiNP i.t. injection. Red arrows point to the high fluorescence point due to IONP-doped-CPNs injection, Figure S5: Biodistribution MRI studies of Endorem^®^. Graphics show the variation in MRI signal intensity (mean ± SEM) in the different organs evaluated: tumor and contralateral healthy brain in the orthotopic glioma model (a) and liver, renal cortex, renal medulla and tumor in the heterotopic GBM model (b), Figure S6: T2 maps and T_2_W images of GBM models injected with Endorem^®^. T2 color-code based maps and T_2_W images acquired before the i.v. administration of Endorem^®^, and at 0.5 and 1 h after the injection, in the of the orthotopic (a) and the heterotopic GBM model (b), numbers indicate the tissues where the ROIs were selected to do the measurements: 1, liver; 2, renal cortex; 3, renal medulla; 4, flank-tumor. Graphics show the variation in T_2_ values (mean ± SEM) of the ROIs along the temporal evaluation in the orthotopic (c), and heterotopic tumor bearing mice (d). Statistical symbols correspond to the comparison of the data to the pre-injection value. (* *p* < 0.05).
