# Supplementary material for: Iron Oxide Incorporated Conjugated Polymer Nanoparticles for Simultaneous Use in Magnetic Resonance and Fluorescent Imaging of Brain Tumors
Source: Pharmaceutics. 2021 Aug 14;13(8):1258. doi: 10.3390/pharmaceutics13081258 (PMC8400017; doi:10.3390/pharmaceutics13081258)
Supplement: Supplementary file 1 [file pharmaceutics-13-01258-s001.zip › pharmaceutics-1319965-supplementary.pdf]

pharmaceutics

Supplementary Material

Iron Oxide Incorporated Conjugated Polymer Nanoparticles for Simultaneous Use in Magnetic Resonance and Fluorescent Imaging of Brain Tumors

N. Arias-Ramos [1], L. E. Ibarra [2,4,*], M. Serrano-Torres [1], B. Yagüe [1], M. D. Caverzán [3,4], C. A. Chesta [3,5], R. E. Palacios [3,5] and P. López-Larrubia [1,*]

Citation: N. Arias-Ramos; L.E. Ibarra; M. Serrano-Torres; B. Yagüe; M.D. Caverzán; C.A. Chesta; R.E. Palacios; P. López-Larrubia Iron Oxide Incorporated Conjugated Polymer Nanoparticles for Simultaneous Use in Magnetic Resonance and Fluorescent Imaging of Brain Tumors. **2021**, *13*, 1258. https://doi.org/10.3390/pharmaceutics13081258

Academic Editor: Pedro Ramos-Cabrer and Jesus Ruiz-Cabello

Received: 14 July 2021
Accepted: 9 August 2021
Published: 14 August 2021

Publisher's Note: MDPI stays neutral with regard to jurisdictional claims in published maps and institutional affiliations.

Copyright: © 2021 by the authors. Licensee MDPI, Basel, Switzerland. This article is an open access article distributed under the terms and conditions of the Creative Commons Attribution (CC BY) license (http://creativecommons.org/licenses/by/4.0/).

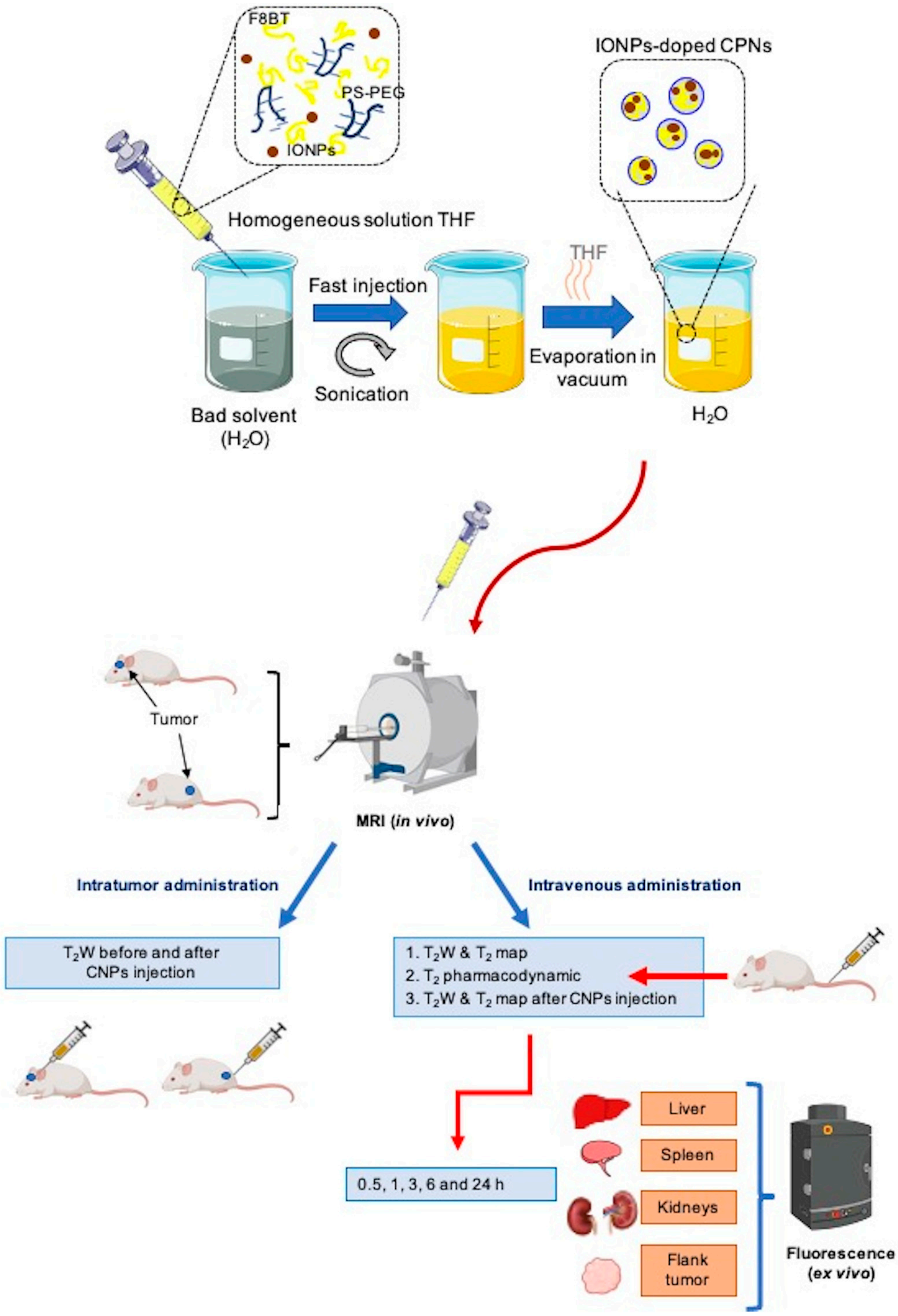

Figure S1. Overview of the experimental workflow followed in the preparation, characterization, in vitro and in vivo evaluation of the IONP-doped CPNs. The scheme shows the steps to synthesize and purified the IONP-doped-CNPs in the upper panels and the preclinical assessment of the nanoparticles in the lower.

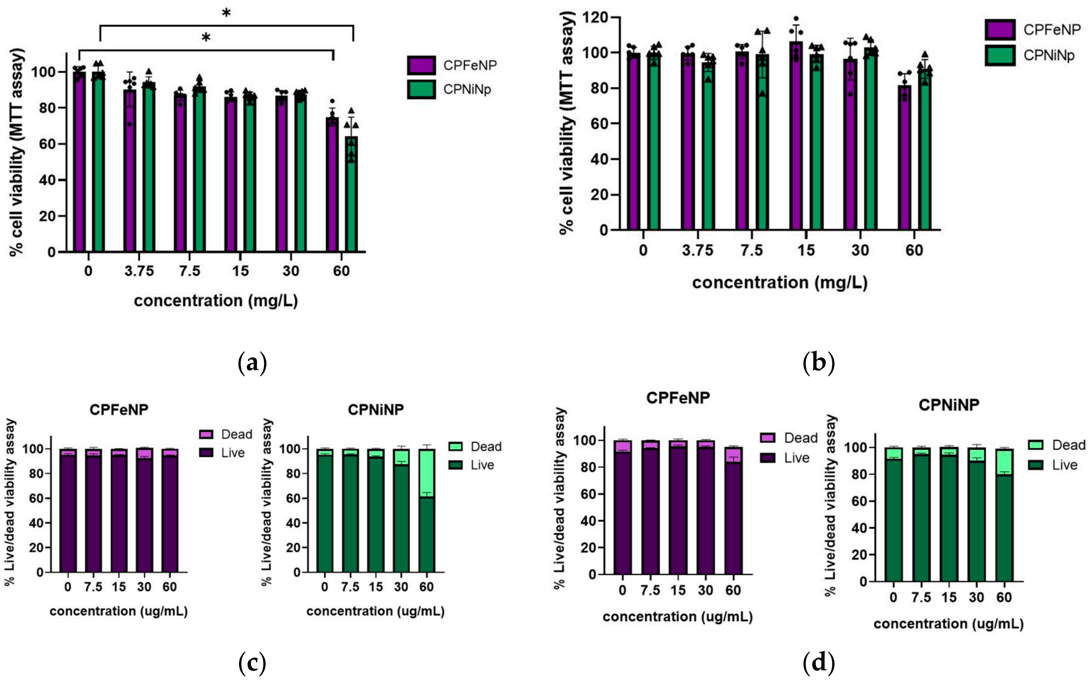

Figure S2. Biocompatibility evaluation of IONP-doped CPNs in GBM human cell lines. MTT viability quantification for (**a**) U-87 MG and (**b**) T98G. Cell viability quantified by flow cytometry using LIVE/DEAD cell viability assays for (**c**) U-87 MG and (**d**) T98G. (*$p < 0.05$).

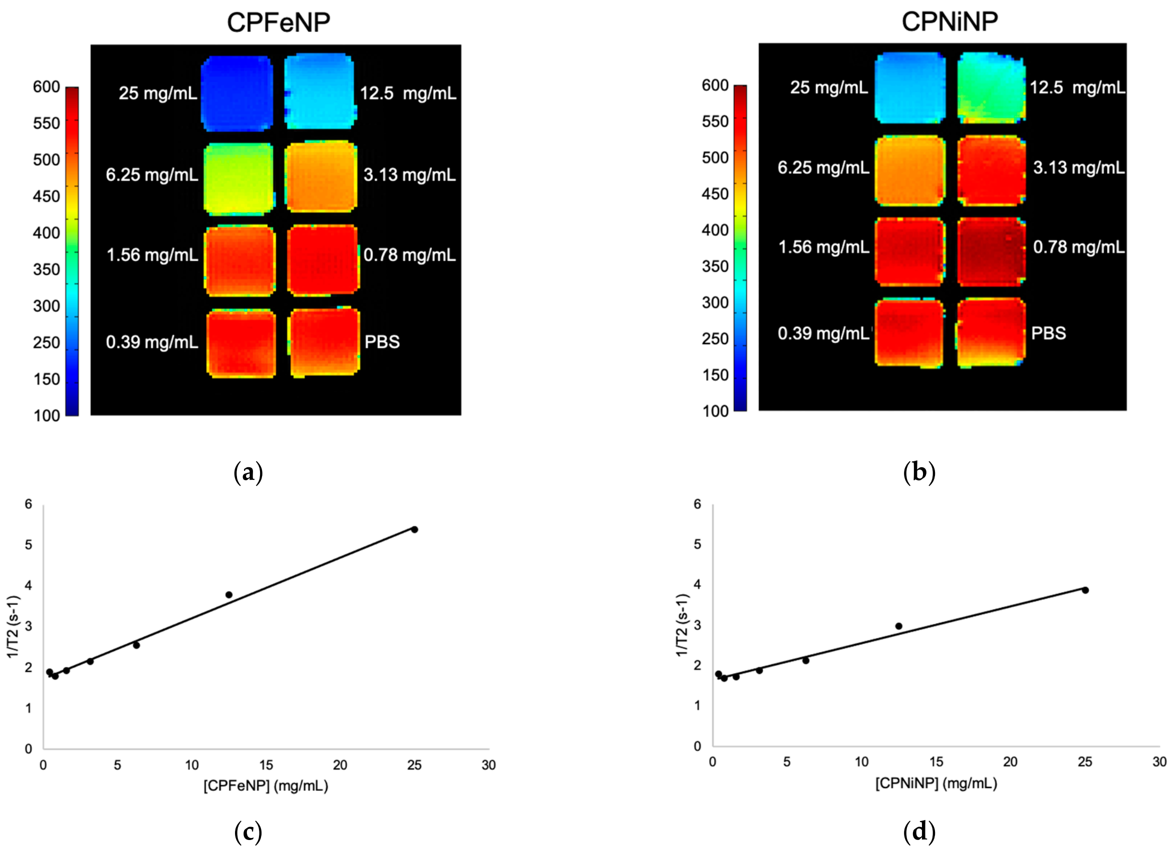

Figure S3. Relaxivities determination of IONP-doped CPNs. T_2 maps of nanoparticles at decreasing concentrations: (**a**) CPFeNP and (**b**) CPNiNP. Graphical linear regression of relaxation rates ($1/T_2$) versus increasing nanoparticle concentration: (**c**) CPFeNP and (**d**) CPNiNP.

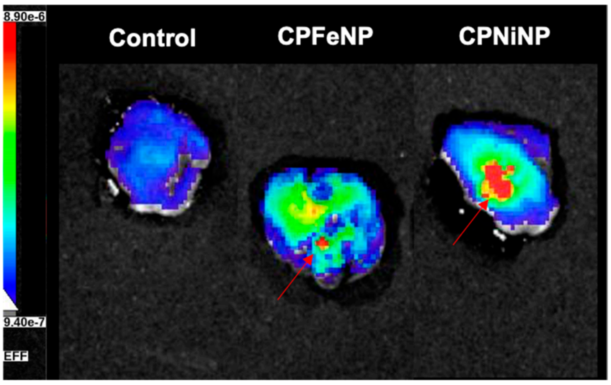

Figure S4. Fluorescence images of tumors after i.t. IONP-doped CPN administration. Images show excised tumors from a control mouse without particle administration (left), a flank tumor after CPFeNP i.t. injection (middle) and a tumor after CPNiNP i.t. injection. Red arrows point to the high fluorescence point due to IONP-doped-CPNs injection.

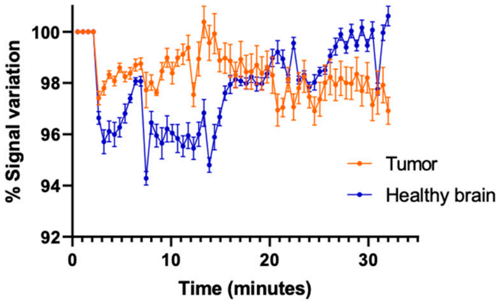

(**a**)

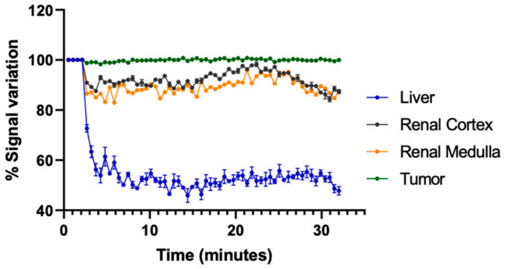

(**b**)

Figure S5. Biodistribution MRI studies of Endorem®. Graphics show the variation in MRI signal intensity (mean ± SEM) in the different organs evaluated: tumor and contralateral healthy brain in the orthotopic glioma model (**a**) and liver, renal cortex, renal medulla and tumor in the heterotopic GBM model (**b**).

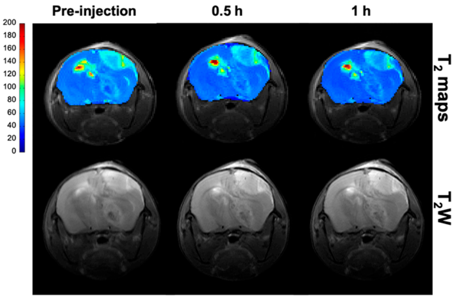

(**a**)

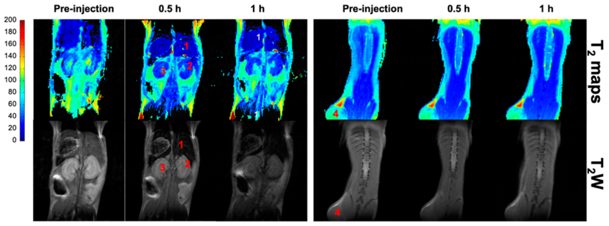

(**b**)

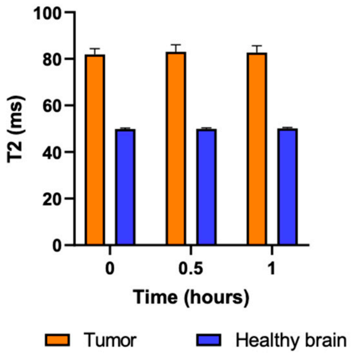

(**c**)

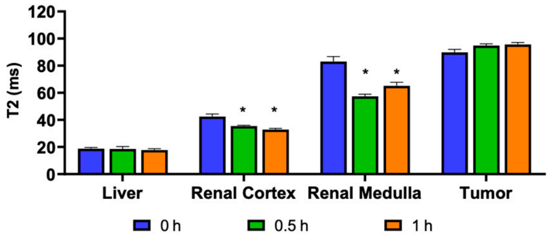

(**d**)

Figure S6. T2 maps and T_2W images of GBM models injected with Endorem®. T2 color-code based maps and T_2W images acquired before the i.v. administration of Endorem®, and at 0.5 and 1 h after the injection, in the of the orthotopic (**a**) and the heterotopic GBM model (**b**), numbers indicate the tissues where the ROIs were selected to do the measurements: 1, liver; 2, renal cortex; 3, renal medulla; 4, flank-tumor. Graphics show the variation in T_2 values (mean ± SEM) of the ROIs along the temporal evaluation in the orthotopic (**c**), and heterotopic tumor bearing mice (**d**). Statistical symbols correspond to the comparison of the data to the pre-injection value. (*$p < 0.05$).
